# Adipose Triglyceride Lipase Loss Promotes a Metabolic Switch in A549 Non–Small Cell Lung Cancer Cell Spheroids

**DOI:** 10.1016/j.mcpro.2021.100095

**Published:** 2021-05-14

**Authors:** Sophie Honeder, Tamara Tomin, Laura Nebel, Jürgen Gindlhuber, Katarina Fritz-Wallace, Maximilian Schinagl, Christoph Heininger, Matthias Schittmayer, Nassim Ghaffari-Tabrizi-Wizsy, Ruth Birner-Gruenberger

**Affiliations:** 1 Diagnostic and Research Institute of Pathology, Medical University of Graz, Graz, Austria; 2 Omics Center Graz, BioTechMed-Graz, Graz, Austria; 3 Faculty of Technical Chemistry, Institute of Chemical Technologies and Analytics, Technische Universität Wien, Vienna, Austria; 4 Otto Loewi Research Center – Immunology and Pathophysiology, Medical University of Graz, Graz, Austria; 5 QPS Austria GmbH, Grambach, Austria; 6 National Center for Tumor Diseases (NCT), Dresden, Germany; 7 German Cancer Research Center (DKFZ), Heidelberg, Germany; 8 Faculty of Medicine and University Hospital Carl Gustav Carus, Technische Universität Dresden, Dresden, Germany; 9 Helmholtz-Zentrum Dresden - Rossendorf (HZDR), Dresden, Germany

**Keywords:** lipolysis, lung cancer, glycolysis, fatty acid metabolism, spheroid, ADPGK, ADP-dependent glucokinase, ATGL, adipose triglyceride lipase, CAM, chorioallantoic membrane, FA, fatty acid, FADS2, fatty acid desaturase 2, FASN, fatty acid synthase, FDR, false discovery rate, GLUT, glucose transporter, HIF, hypoxia-inducible factor, HILPDA, hypoxia-inducible lipid droplet associated, IDH1, isocitrate dehydrogenase 1, KO, knockout, LD, lipid droplet, LFQ, label-free quantitation, MUFA, monounsaturated fatty acid, PANCAN, pan-cancer, SCD1, stearoyl-CoA desaturase 1, TCA, tricarboxylic acid

## Abstract

Cancer cells undergo complex metabolic adaptations to survive and thrive in challenging environments. This is particularly prominent for solid tumors, where cells in the core of the tumor are under severe hypoxia and nutrient deprivation. However, such conditions are often not recapitulated in the typical 2D *in vitro* cancer models, where oxygen as well as nutrient exposure is quite uniform. The aim of this study was to investigate the role of a key neutral lipid hydrolase, namely adipose triglyceride lipase (ATGL), in cancer cells that are exposed to more tumor-like conditions. To that end, we cultured lung cancer cells lacking ATGL as multicellular spheroids in 3D and subjected them to comprehensive proteomics analysis and metabolic phenotyping. Proteomics data are available *via* ProteomeXchange with identifier PXD021105. As a result, we report that loss of ATGL enhanced growth of spheroids and facilitated their adaptation to hypoxia, by increasing the influx of glucose and endorsing a pro-Warburg effect. This was followed by changes in lipid metabolism and an increase in protein production. Interestingly, the observed phenotype was also recapitulated in an even more “*in vivo* like” setup, when cancer spheroids were grown on chick chorioallantoic membrane, but not when cells were cultured as a 2D monolayer. In addition, we demonstrate that according to the publicly available cancer databases, an inverse relation between ATGL expression and higher glucose dependence can be observed. In conclusion, we provide indications that ATGL is involved in regulation of glucose metabolism of cancer cells when grown in 3D (mimicking solid tumors) and as such could be an important factor of the treatment outcome for some cancer types. Finally, we also ratify the need for alternative cell culture models, as the majority of phenotypes observed in 3D and spheroids grown on chick chorioallantoic membrane were not observed in 2D cell culture.

## Highlights

• Adipose triglyceride lipase (ATGL) loss rewires metabolism in lung cancer spheroids.
• Spheroids lacking ATGL grow faster in size and display a pro-Warburg phenotype.
• ATGL loss causes changes in lipid metabolism that may support adaptation to hypoxia.

## Graphical Abstract

**Figure:**
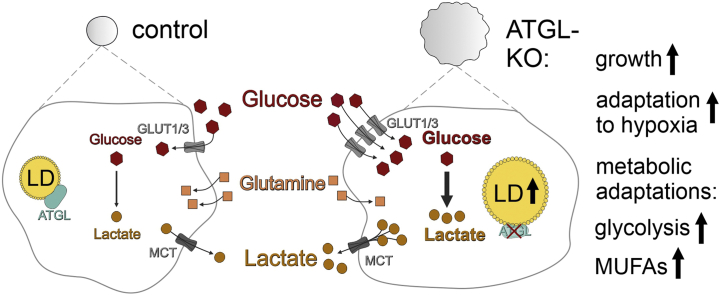

## In Brief

We here demonstrate that loss of adipose triglyceride lipase (ATGL) in an *in-vitro* model of 3D lung carcinoma cell (A549) culture leads to a metabolic switch supporting larger spheroid growth and better adaptation to hypoxia. Cancer spheroids lacking ATGL show a higher abundance of glucose transporters, increased glucose uptake and lactate production, and use alternative lipid metabolic pathways. Overall, our data suggest consideration of ATGL as potentially relevant target in lung cancer.

Malignant transformation is often accompanied by metabolic reprogramming to support anabolic growth (1). The production of biomass for increased proliferation is a priority that is supported by characteristics like switching from oxidative to glycolytic energy production, glutamine-dependent anaplerosis as well as increased *de novo* lipogenesis (1, 2, 3). The more recently emerging changes in lipid metabolism include increased fatty acid (FA) synthesis as well as higher rates of FA uptake by scavenging FA from the tumor environment (4), which highlights the importance of FAs as the main building blocks for membrane synthesis (5). Moreover, accumulation of lipid droplets (LDs) has been recently recognized as characteristic feature in a growing number of cancers (6). However, the role of FA supply from breakdown of intracellular triglyceride stores (from LDs) through lipolysis in cancer cells remains to be elucidated.

Many of the observed cancer-related metabolic phenotypes are highly correlated with hypoxic conditions (7). Limited availability of oxygen (especially in solid tumors) leads to activation of hypoxia-inducible factors (HIFs), which in turn regulate several metabolic pathways (8). HIF-1 is a potent metabolic master regulator, which reduces oxidative metabolism and increases glucose consumption and glycolysis (9, 10). HIF-1 activates glucose transporters (GLUTs) (Glut1 and Glut3), glycolytic enzymes (*e.g.*, hexokinases) as well as proteins facilitating the synthesis and export of lactate such as lactate dehydrogenase and monocarboxylate transporter 4 (11). Furthermore, oxygen availability is required by some enzymes of FA metabolism, such as the fatty acid desaturase stearoyl-CoA desaturase 1 (SCD1) (12). SCD1 facilitates the desaturation of palmitoyl-CoA and stearoyl-CoA generated by *de novo* FA synthesis and thus catalyzes a key step in synthesis of monounsaturated fatty acids (MUFAs), which are crucial for proliferating cells (13, 14, 15). The inactivity of SCD1 under hypoxic conditions highlights the deregulation of lipid metabolism by hypoxia (12).

In addition to FA metabolism deregulations, the accumulation of LDs is associated with hypoxia. Recently, a novel LD associated factor, hypoxia-inducible LD associated protein (HILPDA), was found to promote hypoxic LD formation by inhibiting an enzyme key in breakdown of triglycerides, and thus LDs, namely adipose triglyceride lipase (ATGL) (16). Furthermore, the expression of ATGL protein (but not its mRNA) is reported to be decreased under hypoxic conditions (17), which is mechanistically explained by the activation of HILPDA by HIF-1 (16, 18). Interestingly, we and others have shown that loss of ATGL protein, especially in lung cancer, can lead to a more aggressive cancer phenotype (19) and aid tumor development (20), suggesting a tumor suppressor role of ATGL.

To address the potential metabolic consequences of ATGL loss in cancer, particularly under hypoxic conditions, we investigated ATGL knockout (ATGL-KO) lung cancer cells in *in vivo*–like *in vitro* setups. To this end, we cultured human lung cancer cell lines in 3D, where the oxygen gradient ranges from mild hypoxia to anoxia and thus better mimics the oxygen gradient of avascular solid tumors (21) than in standard 2D cell culture conditions. However, in comparison to tumors *in vivo*, this model does not allow for vascularization. To mitigate this, we have also cultured spheroids on chick chorioallantoic membranes (CAMs), which provide vascularization on the outside of the growing spheroids, mimicking the onset of vascularization in growing tumors.

In both models, we found evidence for an ATGL-dependent metabolic switch that was not observed in cells grown in a monolayer, highlighting the importance of 3D models. Compared with control spheroids, ATGL-KO spheroids grew larger and in a less compact manner, corroborating that ATGL loss could be advantageous for tumor growth. By label-free quantitative proteomics, we identified significant upregulation of a number of proteins involved in glucose or FA metabolism, such as Glut1, a critical glucose transporter, as well as an alternative fatty acid desaturase in ATGL-KO spheroids, which was not observed in 2D cell culture. A stronger glycolytic phenotype as well as increased protein translation was confirmed by label-free quantitative proteomics of ATGL-KO spheroid-derived tumors generated from spheroids cultured on CAM. Our study thus demonstrates a potential pro-Warburg metabolic switch and higher cancer aggressiveness in lung cancer cells upon ATGL loss in *in vivo-*like 3D cell culture models.

## Experimental Procedures

### Experimental Design and Statistical Rationale

According to common standards, the experimental design was chosen to include a minimum of three biological replicates in each group for each experiment. For Western blot, biochemical assays, and microscopy experiments, three biological replicates were used, unless otherwise stated. For these experiments, a two-sided unpaired Students *t* test was performed and group differences considered significant below a threshold of 0.05 (*p* < 0.05 (∗); *p* < 0.01 (∗∗); *p* < 0.001 (∗∗∗)). The data are presented as mean with standard deviation error bars. For proteomics experiments, sample preparation and MS analysis were performed according to section Proteomics Sample Preparation and Analysis and LC-MS/MS Analysis. For spheroid proteomics, 30 spheroids per each biological replicate (n = 3 biological replicates in each group [ATGL-KO or control]) were pooled resulting in six samples. For spheroid-derived tumor proteomics, two tumors derived from spheroids of the same cell line (representing two technical replicates) were analyzed for each biological replicate (n = 3 within each group) resulting in 12 samples. All samples were subjected to label-free quantitation (LFQ), and statistical analysis was performed as described in Data and Statistical Analysis of Proteomics Data*.*

### Reagents

If not stated otherwise reagents were purchased from Sigma-Aldrich.

### Cell Culture

A549 lung adenocarcinoma cells were obtained from CLS and cultured at 37 °C and 5% CO_2_ in glutamine-free Roswell Park Memorial Institute-1640 (RPMI-1640, R0883) medium with phenol-red and 2 g/l of glucose, additionally supplemented with 10% fetal bovine serum (Gibco) and 146 mg L-glutamine (Gibco). Knockout of *PNPLA2*, the gene encoding for ATGL, was achieved by CRISPR-Cas9 methodology, as described previously (19). Briefly, A549 parental cells were transfected with either a plasmid for directed knockout of human ATGL (sc-401711; Santa Cruz Biotechnology) or a control plasmid (sc-418922; Santa Cruz Biotechnology) using Lipofectamine3000, generating ATGL-KO or corresponding control cells. The cells were single cell selected by either fluorescence-activated cell sorting or serial dilution to obtain single cell clones that were further expanded and checked for ATGL protein and mRNA expression.

### 3D Cell Culture

For the generation of multicellular tumor spheroids, the cells were seeded at 10,000 cells per well in 100 μl of medium into an Ultra-Low Attachment 96-well Plate (Corning). Spheroid formation was induced by centrifugation at 1200 rpm for 20 min. The cells were kept in culture for a minimum of 10 days, and additional 50 μl of medium was supplemented twice during the cultivation.

### Chorioallantoic Membrane Assay

CAM assay was carried out using the *ex ovo* CAM method according to Deryugina and Quigley (22). The *ex ovo* cultivation is more readily accessible for *in situ* analysis than the *in ovo* CAM method and allows an increased number of on-plants per embryo (22). Briefly, fertilized white Lohmann chicken eggs were cleaned and incubated under regular rotation at 37 °C and 60% humidity (Incubator Easy200, J.Hemel Brutgeräte). The egg shell was cracked on day 3 of embryonic development and again incubated in a sterile dish at 37 °C and 60% humidity. On day 9 of embryonic development, precultivated (10 days in 3D culture) A549 ATGL-KO and control spheroids were grafted on the CAM. Per each egg, three on-plant areas were allocated, and per each on-plant, five spheroids of the same cell line were seeded. To reduce potential egg-driven bias, ATGL-KO and control on-plants were randomized on different eggs. Chick embryos were further incubated for another 5 days during which small spheroid derived tumors were formed. Five days later, on-plants with tumors were harvested, washed, and snap frozen. Two on-plants per each ATGL-KO (n = 3) and control (n = 3) cell line were used for proteomics analysis.

### Proteomics Sample Preparation and Analysis

Lysis of spheroids from 3D culture (30 per each individual ATGL-KO clone [n = 3] or control clone [n = 3] of A549 cells) as well as of spheroids-derived small tumors grown on CAM was carried out in lysis buffer (100 mM Tris pH = 8, 1% sodium dodecyl sulphate, 10 mM tris(2-carboxyethyl) phosphine, 40 mM chloroacetamide), followed by several sonication cycles. Protein content was estimated using bicinchoninic acid assay (Thermo Fisher Scientific) after which 100 μg of protein per each sample was acetone precipitated overnight. The following day, protein pellets were re-dissolved in 25% trifluoroethanol (in 100 mM Tris pH = 8.5), diluted to 10% trifluoroethanol with ammonium bicarbonate, and digested for 2 h with LysC (Thermo Fisher Scientific) then overnight with trypsin (Thermo Fisher Scientific). Finally, samples were diluted in running buffer A (0.1% formic acid, 5% acetonitrile; running buffer B: pure acetonitrile containing 0.1% formic acid), and 1 μg of sample was injected for LC-MS/MS analysis.

### LC-MS/MS Analysis

3D spheroid cell culture samples were concentrated on an enrichment column (C18, 5 μm, 100 Å, 20 × 0.1 mm, Thermo Fisher Scientific) for 6 min with the flow rate of 5 μl/min. Chromatographic separation was carried out on an Acclaim PepMap RSLC C18 nanocolumn (2 μm, 50 × 75 μm) (Thermo Fisher Scientific) at 60 °C with the flow rate of 0.3 μl/min and the following gradient: 6 to 154 min: 4 to 25% B, 154 to 159 min: 25 to 95% B, 159 to 169 min: 95% B, 169.1 to 184 min: 4% B.

For spheroids cultured on CAM, chromatographic separation was carried out on an Aurora Series UHPLC C18 column (250 mm × 75 μm, 1.6 μm) (Ionopticks) with the flow of 0.25 μl/min and the following gradient: 0 to 18 min: 2% B, 18 to 160 min: 2 to 25% B, 160 to 167 min: 25 to 35% B, 167 to 168 min: 35 to 95% B, 168 to 178 min: 95 to 2% B. Mass spectrometry was performed on Orbitrap Velos Pro (Thermo Fisher Scientific) operated in positive ion mode by alternating full scan MS (m/z 300–2000, 60,000 resolution) in the ion cyclotron resonance cell and MS/MS by collision-induced dissociation of the ten most intense peaks in the ion trap (with dynamic exclusion enabled for 35 s). The mass spectrometry proteomics data were deposited to the ProteomeXchange Consortium *via* the PRIDE (23) partner repository with the data set identifier PXD021105 and doi:10.6019/PXD021105.

### Data and Statistical Analysis of Proteomics Data

For both data sets, data analysis, database search, and quantitation were carried out by MaxQuant (v1.6.14.0) (24) software. For statistical analysis, Perseus (v1.6.13.0) (25) was employed. For both data sets in the search criteria false discovery rate (FDR) for peptide, peptide-to-spectrum as well as protein matches was set to 1%. Peptide tolerance was set to ±20 and ±4.5 for the first and main peptide search, respectively. Product mass tolerance was set to ±0.5 Da. Cysteine carbamidomethylation was set as static, whereas methionine oxidation and N-terminal acetylation were set as dynamic modifications. Minimum required peptide length was six amino acids and maximum number of allowed tryptic mis-cleavages two. No intensity threshold for individual spectra was defined. Max Quant spectral files were uploaded to MS-Viewer (https://msviewer.ucsf.edu/) with the following accession keys: octvhdisxq for the 3D spheroid data set and vaqbljjeke for the 3D spheroids on CAM data set.

#### Spheroid Proteomics Data Analysis

For 3D spheroids data, SwissProt human fasta file (downloaded on 16.04.2019, 20,467 sequences) containing most common protein contaminants was used as a database. Protein quantitation was based on LFQ, with a minimum of two peptides per protein (unique and razor) as quantitation requirement. Match between runs was enabled in the retention time window of 1 min and alignment window of 20 min (respectively).

This resulted in a list of 2569 proteins with their corresponding LFQ values. LFQ values were log_2_ transformed (resulting in invalid values [NaN] for any missing value), and contaminants were removed. The matrix was then filtered to contain at least three valid values (LFQ intensities >0 prior transformation) in at least one group (control or ATGL–KO) to exclude proteins that were not identified across all samples of one group. This reduced the matrix to 1573 proteins, and remaining missing values were imputed from normal distribution (downshift 2, width 0.3) (supplemental Table S1). Consequently, Student *t* tests were performed with the following criteria: *p*-value of at least 0.05, S0 of 0.5 (roughly corresponds to a minimum fold change of 1.5) and permutation-based FDR set to 5% to correct for multitesting. Histograms of data distribution per each sample including the quantile-quantile (qq) plots of the most prominent significantly altered proteins is displayed in supplemental Fig. S1.

#### CAM Spheroid Proteomics Data Analysis

For data analysis of CAM spheroid-derived small tumors (n = 3 biological replicates per condition, *i.e.*, derived from three singe cell derived ATGL-KO or control clones), data from duplicates of on-plants per each biological replicate (two individual CAM derived tumors from the same ATGL-KO or control clone) were combined by configuring them as fractions in MaxQuant. Owing to mixed species sample origin (human cell line and the chicken membrane), in addition to the human database containing contaminants, data were also searched against the TrEMBL chicken database (downloaded on 12.03.2019, 41,601 sequences). Search parameters were set as described above. For LFQ analysis, a minimum of two peptides was required for quantitation with only unique peptides allowed. In this way, the possibility of errors in quantitation due to false assignment was reduced. Finally, the result output was filtered to contain only proteins uniquely assigned to human. LFQs of all individual human proteins were then normalized on the sum of LFQs of all quantified human proteins per each sample.

The resulting list of 1778 protein groups with normalized LFQ values was imported into Perseus, where the matrix was log_2_ transformed, and additional contaminants were removed, and the matrix was filtered to contain at least three valid values in at least one group, reducing the matrix to 620 proteins (supplemental Table S2). Missing values were then imputed from the normal distribution of the data after downshift of 2 and setting the width to 0.3, and Student *t* tests were performed with a significance threshold for FDR corrected *p*-value <0.05 and S0 value set to 0.5. Distribution of data points from CAM spheroid data set is also included in the supplemental Fig. S1.

### Reactome Pathway Enrichment

Proteins higher expressed in the ATGL-KO spheroids grown on CAM with a fold change greater than two were used for pathway enrichment analysis using String functional protein database (https://string-db.org/, v11.0). FDR corrected *p*-value for significance was <5%.

### Western Blot

Cells were harvested in 1× CST lysis buffer (Cell Signaling Technology), and protein quantification was performed using a Pierce BCA Protein Assay kit (Thermo Fisher Scientific) with BSA as standard. Proteins (15 μg protein from cell lysates, unless otherwise stated) were separated on precast NuPAGE 4 to 12% Bis-Tris Midi Protein Gels (Invitrogen). Proteins were blotted onto a nitrocellulose membrane (Amersham) followed by blocking of unoccupied sites on the membrane by 5% skim milk in Tris-buffered saline containing 0.1% Tween 20 (TBS-T). Primary antibodies against Glut1 (ab128033, Abcam) and Glut3 (sc-74497, Santa Cruz Biotechnology) were diluted 1:1000 in 5% skim milk; primary antibody against β-actin (A5441, Sigma Aldrich) was diluted 1:5000 in 5% skim milk. Membranes were incubated with primary antibodies at 4 °C overnight. After washing with TBS-T three times, membranes were incubated for 1 h at room temperature with diluted (1:5000) secondary antibody in TBS-T: anti-rabbit horseradish peroxidase (HRP)-linked antibody (Cell Signaling Technology) for Glut1 and β-actin and anti-mouse HRP-linked antibody for Glut3 blots. Visualization of antibodies bound to proteins was achieved by incubation with Pierce ECL (Thermo Fisher Scientific) and detection on a ChemiDoc System (Bio-Rad).

### Lactate Production/Glucose and Glutamine Consumption

For the assessment of lactate production as well as glucose and glutamine consumption, bioluminescent assays were employed (Lactate/Glucose/Glutamine&Glutamate Glo Assay, Promega) and carried out according to manufacturer’s instructions on a LumiStar luminometer (BMG Labtech). Briefly, cells were seeded at a concentration of 10,000 cells in 100 μl of medium into the wells of a 96-well plate (Corning) for 2D and into the wells of an Ultra-Low Attachment 96-well Plate (Corning) for 3D cell culture. In 2D cell culture experiments, aliquots of culture medium on the cells were removed 1, 2, and 3 days after seeding, and cells were harvested on day 3 after seeding. In spheroid experiments, medium was added to the cells on days 3 and 6 after seeding, and culture medium aliquots were removed on day 1, 7, 8, 9, 10 (and 13) after seeding, and cells were harvested on day 10 (or day 13, respectively). In each measurement, calibration curves with lactate or glucose standards of known concentration were included, as well as a dilution of culture medium as a control and PBS as a blank. The concentrations of lactate or glucose in the culture medium were calculated based on the corresponding calibration curves. For glutamine assessment, one aliquot of each sample was treated with glutaminase, which converted all glutamine into glutamate, whereas another aliquot was treated with buffer only. All samples were then mixed with an equal volume of glutamate detection solution (including the glutamate calibration curve) followed by incubation for 1 h at room temperature before measurement. Glutamate concentration was determined based on the calibration curve, and glutamine was assessed by subtraction of glutamate concentrations of samples not treated with glutaminase from samples that were treated with glutaminase.

### Spheroid Dissociation and Lipid Droplet Analysis

For dissociation of spheroids to obtain single spheroid-derived cells, five spheroids were pooled and washed with PBS. One hundred fifty microliter Accutase cell detachment solution (Merck Millipore, #SCR005) was added, followed by careful pipetting. The spheroid solution was incubated for 10 min in Accutase solution followed by another round of pipetting, resulting in a cell suspension.

For LD analysis, the obtained cell suspension was seeded into glass-bottom cellview cell culture dishes (Greiner bio one, #627965) and allowed to re-attach for 4 h. The cells were washed with PBS and incubated in 1:1000 BODIPY 493/503 (Invitrogen, #D3922) in culture medium for 10 min at 37 °C. Cells were washed twice with warm PBS and fixed by incubation with warm 3.7% formaldehyde solution for 10 min at 37 °C. Cells were washed again and mounted in 25 μl Vectashield Antifade Mounting Medium with DAPI (Vector Laboratories, #H-1200-10). LD imaging and volume analysis was performed as described previously (19). Briefly, images were acquired on a Nikon A1+ confocal laser scanning microscope and analyzed with ImageJ. LD volume was quantified and normalized to cell count. Average LD volume per cell was calculated from three ATGL-KO clones and three control clones from a total of 76 or 80 cells, respectively.

### Fatty Acid Methyl Ester Extraction and Measurement

Five spheroids per each ATGL-KO or control clone were pooled, washed with cold PBS, and dissociated in 150 μl Accutase solution followed by addition of 100 μl H_2_O, 300 μl methanol, and 1 ml methyl-tert-butyl ether together with 1 μg of a methyl pentadecanoate (C15:0) internal standard (Sigma Aldrich, #76560). The samples were sonicated for 10 min to dissolve membranes and extract lipids, followed by phase separation through centrifugation at 2000*g* for 10 min. The organic phase was transferred to a new tube, and the aqueous phase was re-extracted with 120 μl H_2_O, 120 μl methanol, and 400 μl methyl-tert-butyl ether. The organic phases were combined and dried under a N_2_ stream. The total lipid extracts were hydrolyzed, and the FAs further esterified in 200 μl 0.1 M sodium hydroxide (NaOH) in methanol for 10 min at 80 °C and 5 min on ice followed by addition of 200 μl boron trifluoride (Sigma Aldrich, #B1252) and further incubation for 10 min at 80 °C and 5 min on ice. FA-methylesters were extracted by addition of 200 μl saturated NaCl and 400 μl cyclohexane and overhead rotation for 5 min. Phases were separated by centrifugation (2000*g*, 5 min), and the cyclohexane phase was transferred to a new tube. The samples were re-extracted with 200 μl cyclohexane, and the cyclohexane phases were combined and dried under stream of N_2_. The samples were re-dissolved in 200 μl dichloromethane and further diluted 1:5 before measurement. FA-methylesters were measured with a Scion 436-GC system (Bruker) coupled to an amaZon speed ETD ion trap mass spectrometer (Bruker). One microliter of each sample was injected, and a split mode of 1:20 was applied with the inlet temperature set to 280 °C. Helium was used as the carrier gas with a flowrate of 1 ml/min. The oven temperature was ramped from 50 °C to 300 °C after 1 min with a rate of 20 °C/min and held at 300 °C until 30 min. The mass spectrometer was operated in positive ion mode, and the mass range between 100 and 1000 m/z was scanned. The raw data were analyzed with Lipid Data Analyzer (version 2.7.0), and FA species were normalized to an internal standard (methyl pentadecanoate) and protein content per spheroid. Furthermore, FA ratios or abundances were normalized to the control group.

### Histology of Spheroids

For histological analysis, spheroids were harvested, followed by fixation with 4% paraformaldehyde overnight. The following day, dehydration was carried out with an ethanol series, and toluene was used for clearing. Tissue was immersed with infiltration paraffin, followed by embedding. Wax blocks were stored at 4 °C until sectioning. Blocks were cut in 5 μm thick sections with the semiautomated rotary microtome RM2245 (Leica Biosystems). For immunohistochemistry staining, the sections were deparaffinized and rehydrated, followed by treatment with antigen retrieval solution. The sections were then incubated with 1:50 diluted Ki67 specific primary antibody (Dako, Agilent) followed by HRP-bound secondary antibody specific for the primary antibody (Dako, Agilent). Detection was carried out by HRP/3,3′-diaminobenzidine detection kit (Ultravision, Thermofisher). Pictures of stained sections were taken with the Olympus BX53 using the Olympus cellSens Dimension 1.18 software (Olympus Corporation).

### Spheroid Imaging

Cells were seeded for 3D culture as described above. The cell culture plates were placed in an Okolab stage top incubator mounted on a Nikon TiE-2 microscope equipped with an Andor Zyla 4.2sCMOS camera. Using the JOBS module of the imaging software NIS Elements (V5.20.01), an automated acquisition program was established to scan the spheroid containing wells. For each of those wells, a brightfield z-stack was acquired, range 600 μm and 30 μm step size, at 100× magnification. The NIS Elements General Analysis 3 tool was used to perform an automated analysis for size and shape (including area (*A*) and perimeter estimation). For this the z-stack was reduced by selecting the best focus plane followed by a thresholding step with additional smoothing and size exclusion to remove any in or out of focus precipitate. The circularity of spheroids was estimated with the following formula: $Circularity=\;4\pi A/perimeter^{2}$, where a circularity of 1 represents a perfect circle and the value decreases as the values for the perimeter increase.

## Results

### A549 ATGL-KO Spheroids Grow Larger in Size Than Control Spheroids

**Fig. 1:**
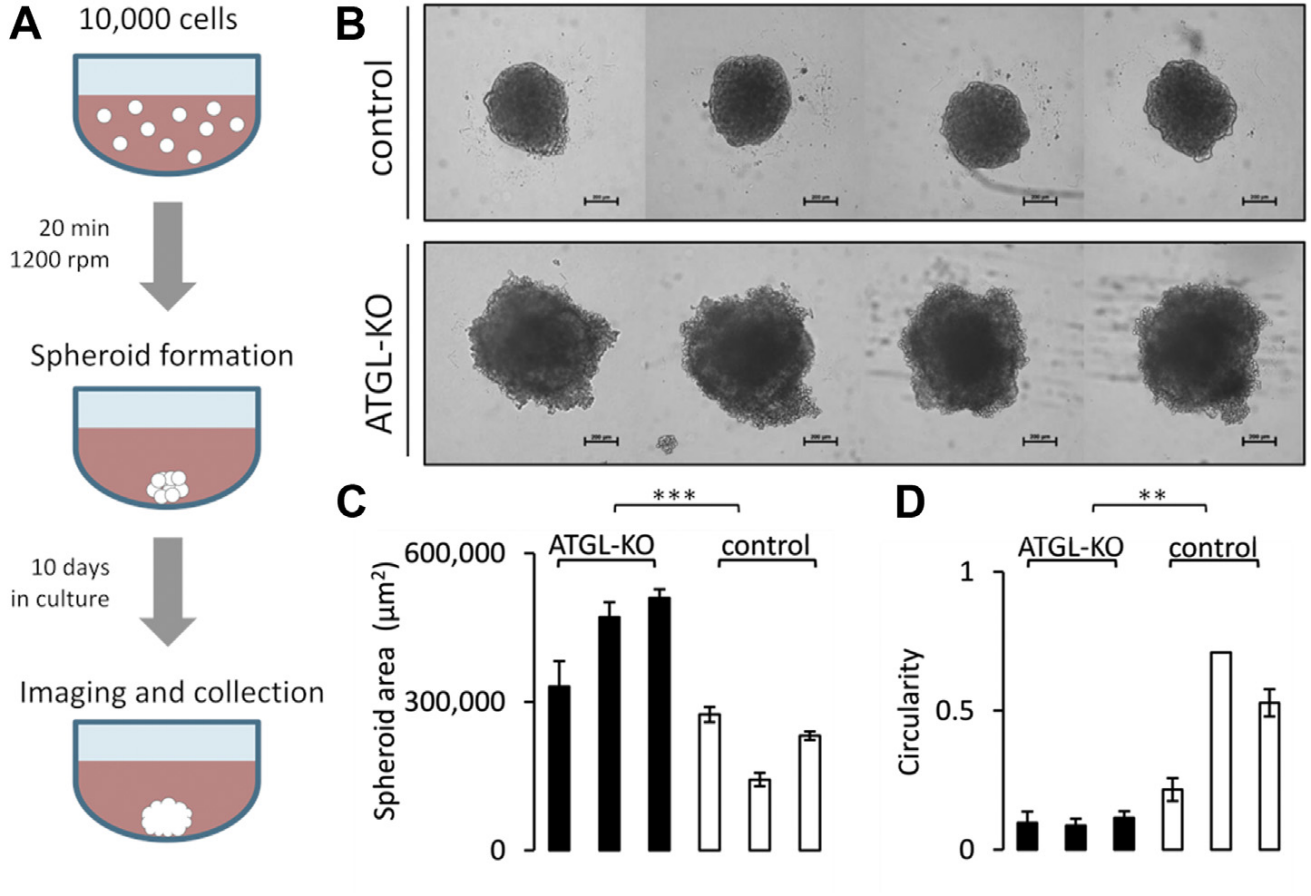
**Workflow, size, and morphology of ATGL-KO and control cells in 3D culture.***A*, workflow for spheroid preparation; spheroids were imaged at regular intervals during 10 days of incubation. *B*, representative images of control (*upper panel*) and ATGL-KO (*lower panel*) spheroids after 10 days in culture (100× magnification, 200 μm scale bar). *C* and *D*, ATGL-KO spheroids grew significantly bigger in size compared with control and were less circular in shape (maximum circularity would be 1; n = 3 biological replicates per group and n = 4 spheroids per replicate), one of three individual experiments shown; ∗∗*p* < 0.01; ∗∗∗*p* < 0.001. ATGL, adipose triglyceride lipase; ATGL-KO, ATGL knockout.

**Fig. 2:**
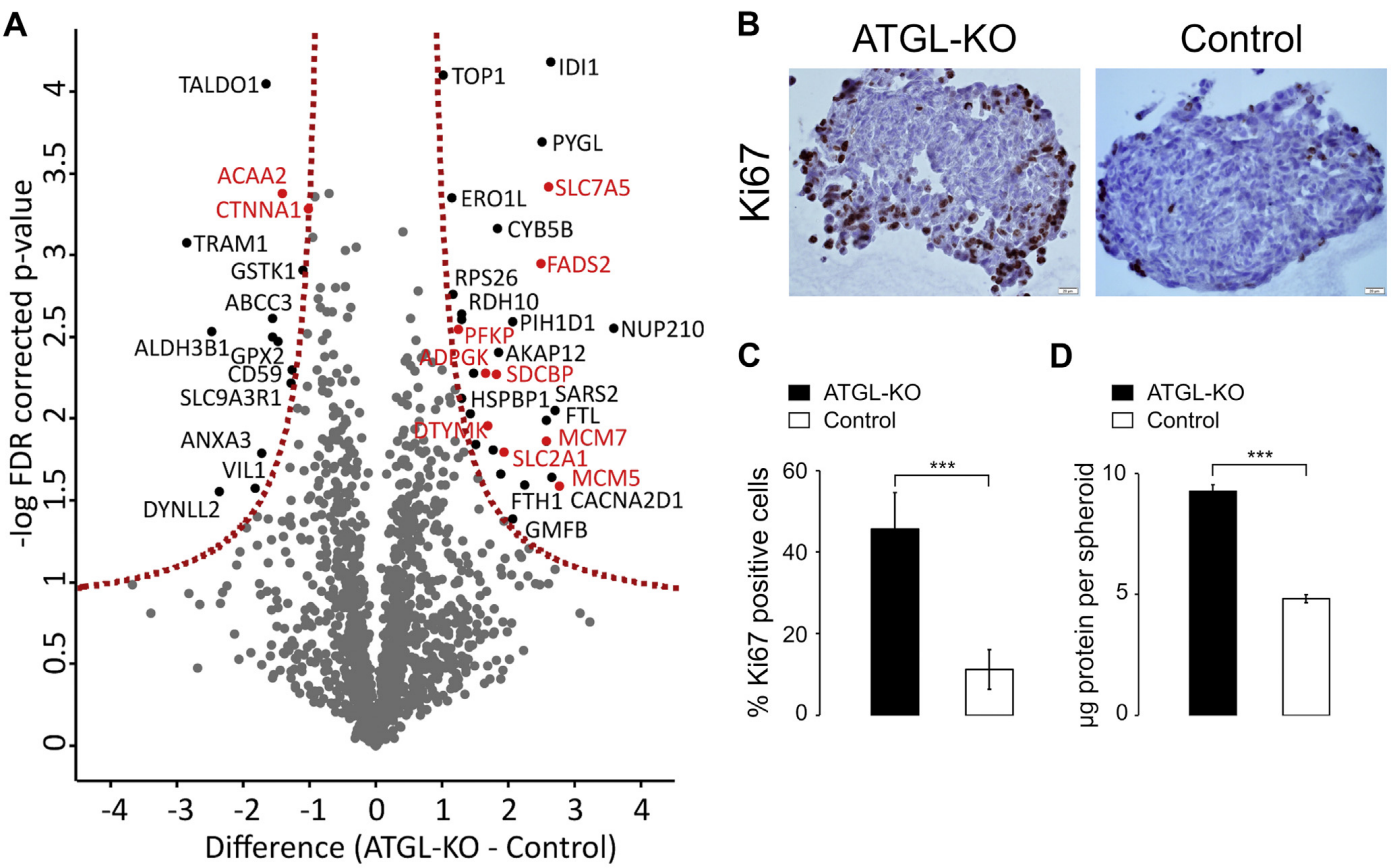
**Label-free quantitation (LFQ) of ATGL-KO and control spheroids**. *A*, volcano plot of the LFQ data after filtering for at least three valid values in at least one group; significance threshold Student *t* test *p*-value 0.05, FDR 5%, S0 0.5; proteins marked in *red* are addressed in more detail in the text. *B*, immunohistochemical (IHC) analysis using monoclonal Ki67 antibody (mitosis marker) (200× magnification). In the *left image* (ATGL-KO cells), more cells were stained with mitosis marker, suggesting a highly proliferative outer rim of the spheroid; *C*, quantification of Ki67 stained cells in percent represented as % Ki67 positive cells; *D*, protein concentration per spheroid, data shown from three individual experiments (n = 3 biological replicates); ∗∗∗*p* < 0.001. ATGL, adipose triglyceride lipase; ATGL-KO, ATGL knockout.

Three-dimensional cell culture can provide an *in vitro* environment that is more similar to physiological tumor conditions, in which some cells experience hypoxia, low nutrient availability, as well as low extracellular pH (21, 26). Three-dimensional cell culture can be achieved by growing cells as spheroids rather than as an attached monolayer. We cultured A549 parental, control and ATGL lacking (ATGL-KO) cells as spheroids using Corning Costar Ultra-Low Attachment plates. Ten thousand cells were seeded per well, and spheroid formation was facilitated by centrifugation (Fig. 1*A*). We assessed spheroid growth by imaging the cells over a period of 10 days, representative pictures shown in Figure 1*B* and supplemental Fig. S2*A*. While A549 control spheroids show a similar growth curve than A549 parental spheroids, ATGL-KO spheroids grow significantly faster (supplemental Fig. S2*B*). The spheroids lacking ATGL were significantly larger in size than the respective control cells after growth of 10 days (Fig. 1*C*). To demonstrate that ATGL-KO spheroids are not only larger in size but do contain more cells, we determined the amount of protein in spheroids. We found that protein content is significantly higher in ATGL-KO spheroids (Fig. 2*D*), indicating higher number of cells per spheroid and therefore increased growth, which was also confirmed by single cell count through flow cytometry (supplemental Fig. S3*B*). In addition, the morphology of ATGL-KO spheroids proved to be different from control spheroids in that they grew less circular and showed more single cellular outgrowths, whereas control spheroids depicted a smoother surface (Fig. 1, *B* and *D*).

While we previously reported that there was no growth difference between ATGL-KO and control cells grown in a 2D monolayer when cultured in F12 K medium (N3520, 1.26 g/l glucose) (19), we observed that ATGL-KO cells did grow significantly faster also in 2D when cultured in higher glucose containing medium (RPMI 1640, R0883, 2 g/l glucose) (supplemental Fig. S4*A*). However, in 3D cell culture, where the higher glucose medium is used as well, the growth difference was more pronounced (Fig. 1, *B* and *C*).

### Upregulated Glycolytic and Fatty Acid Metabolism–Related Proteins in ATGL-KO A549 Spheroids

To identify possible proteomic changes associated with the observed growth difference, we analyzed the proteome of ATGL-KO and control A549 spheroids by LFQ proteomics. As a result, we identified a number of proteins that differed in abundance between the two groups (ATGL-KO and control) (Fig. 2*A*, supplemental Table S1).

Interestingly, several of the proteins with higher abundance in ATGL-KO spheroids were associated with a more glycolytic phenotype. These include glucose transporter 1 (Glut1, *SLC2A1*, P11166), a GLUT with high affinity for glucose and a HIF-1 target, which is associated with poor prognosis in many tumors including lung cancer (11, 27, 28). Other glucose metabolism–related proteins upregulated in ATGL-KO spheroids included ADP-dependent Glucokinase (*ADPGK*, Q9BRR6), as well as ATP-dependent 6-phosphofructokinase (*PFKP*, Q01813). ADPGK is expressed in many tumors and phosphorylates glucose to glucose-6-phosphate in dependence of ADP and thus catalyzes the first reaction of glycolysis (29), whereas PFKP catalyzes the irreversible conversion of fructose 6-phosphate to fructose-1,6-bisphosphate, the rate limiting reaction of glycolysis. These proteins indicate a more glycolytic phenotype and hence a potential pro-Warburg metabolic shift.

Next to glycolytic enzymes, we observed a prominent upregulation of several proteins of the minichromosome maintenance protein complex (*MCM3*, P25205; *MCM5*, P33992; and *MCM7,* P33993), which is a key complex for DNA replication, indicating higher rates of cell division in ATGL-KO spheroids (30). This was also indicated by higher abundance of thymidylate kinase (*DTYMK*, P23919), a key enzyme in pyrimidine and thus DNA synthesis (31). The large amino acid transporter LAT1 (*SLC7A5*, Q01650) was found increased in ATGL-KO spheroids enabling the transport of large neutral amino acids such as tyrosine, leucine, isoleucine, valine, and phenylalanine into the cell (32). Increased uptake of amino acids could imply higher protein production. An increase in production of biomass in ATGL-KO spheroids was corroborated by protein estimation of ATGL-KO and control spheroids, which demonstrated that ATGL-KO spheroids had a 2-fold higher protein content (Fig. 2*D*). Immunohistochemical staining of the different spheroids (ATGL-KO and control) with antibody against the mitosis marker Ki67 additionally supported a more proliferative phenotype of ATGL-KO spheroids (Fig. 2, *B* and *C*).

In addition, we observed changes in lipid metabolism proteins such as fatty acid desaturase 2 (*FADS2*, O95864), which was higher abundant in ATGL-KO spheroids. FADS2 was recently recognized as essential salvage pathway for FA desaturation in an event of SCD1 inhibition, which is commonly the case in hypoxia (33). In the ATGL-KO spheroids, we further identified a protein associated with invasiveness of several types of cancer: Syntenin-1 (*SDCBP*, O00560). This adaptor protein was shown to increase migration and invasiveness of cancers through different pathways (34, 35). The relatively strong morphological difference of ATGL-KO and control spheroids (Fig. 1*B*) might be because of differences in migratory potential of the cell lines, as previously published by us (19).

Among the proteins with lower abundance in ATGL-KO spheroids, we identified catenin alpha-1 (*CTNNA1*, P35221), a tumor suppressor known to be downregulated in a number of human cancers (36, 37). Owing to its function in the E-cadherin-catenin complex, α-catenin is important for cell-cell adhesion and thus tissue organization (38). Its lower abundance in ATGL-KO spheroids might contribute to the observed changes in spheroid morphology such as lower circularity and a rougher, more ragged-looking outer surface of the spheroids when compared to controls (Fig. 1*B*). Moreover, we identified 3-ketoacyl-CoA thiolase (*ACAA2*, P42765) in the group of downregulated proteins in ATGL-KO spheroids. 3-ketoacyl-CoA thiolase is an enzyme involved in the mitochondrial beta oxidation pathway, an aerobic process in which energy is produced from FAs (39). This could indicate that energy production through glycolytic processes is favored over (FA) oxidative pathways in ATGL-KO spheroids.

### Increased Glucose Uptake in A549 Spheroids Lacking ATGL

**Fig. 3:**
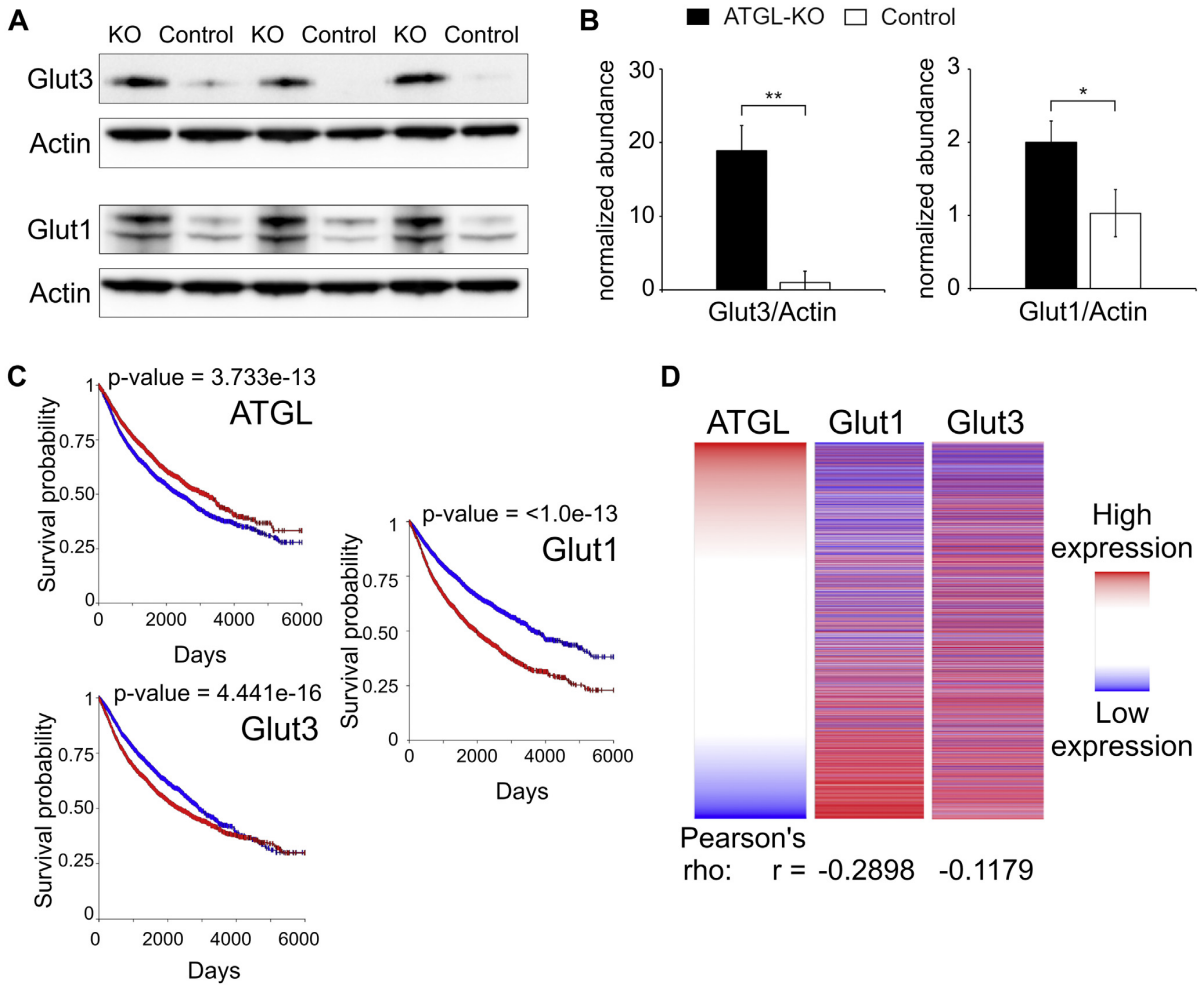
**ATGL-KO spheroids show higher expression of glucose transporters compared with ATGL-control spheroids**. *A*, Western blot of Glut3 and Glut1 with actin from the same membranes as loading control. *B*, quantification of bands from Western blot (3D cells): represented as abundance normalized to the mean of the control group (control set to 1; n = 3 biological replicates). *C*, Kaplan-Meier plot of ATGL, Glut1, and Glut3 using a TCGA PANCAN data set. The plots were generated using the Xena Functional Genomics Explorer. *D*, gene expression correlation of ATGL with Glut1 and Glut3 on an all-cancer TCGA data set (PANCAN). The plot was generated using the Xena Functional Genomics Explorer; ∗*p* < 0.05; ∗∗*p* < 0.01. ATGL, adipose triglyceride lipase; ATGL-KO, ATGL knockout.

To verify the increased expression of GLUTs in ATGL-KO spheroids, we performed immunoblotting of GLUTs Glut1 and Glut3. Both GLUTs were confirmed to be significantly higher expressed in ATGL-KO compared with control cells (Fig. 3, *A* and *B*). We assessed Glut1 and Glut3 content in ATGL-KO and control cells in 2D culture as well and found less pronounced (non-significant) changes compared with the changes observed in 3D culture (supplemental Fig. S4, *B* and *C*). As both ATGL and Glut1/3 are regulated as a response to hypoxia (16, 17, 40, 41), we were wondering if there was a general regulatory link between these proteins in cancer. To this end, we employed the Xena Functional Genomics Explorer (https://xenabrowser.net/) to address ATGL (gene name *PNPLA2*) and Glut1/3 (gene names: *SLC2A1/3*) gene expression in a large all cancer (PANCAN) data set from The Cancer Genome Atlas (TCGA; n = 12,839 samples). Intriguingly, we observed an inverse correlation between ATGL and Glut1 RNA abundance (Fig. 3*D*; Person’s r = −0.2898) and also, although less prominent, between ATGL and Glut3 RNA (Fig. 3*D*, r = −0.1179). The inverse correlation between ATGL and Glut1/3 expression in cancer was further corroborated by Kaplan-Meier survival plots on the same PANCAN data set. While higher expression levels of Glut1 and 3 showed worse outcome in cancer patients, for ATGL it was the opposite (Fig. 3*C*). Similar observations were made for the TCGA lung adenocarcinoma data set (n = 706; supplemental Fig. S5).

**Fig. 4:**
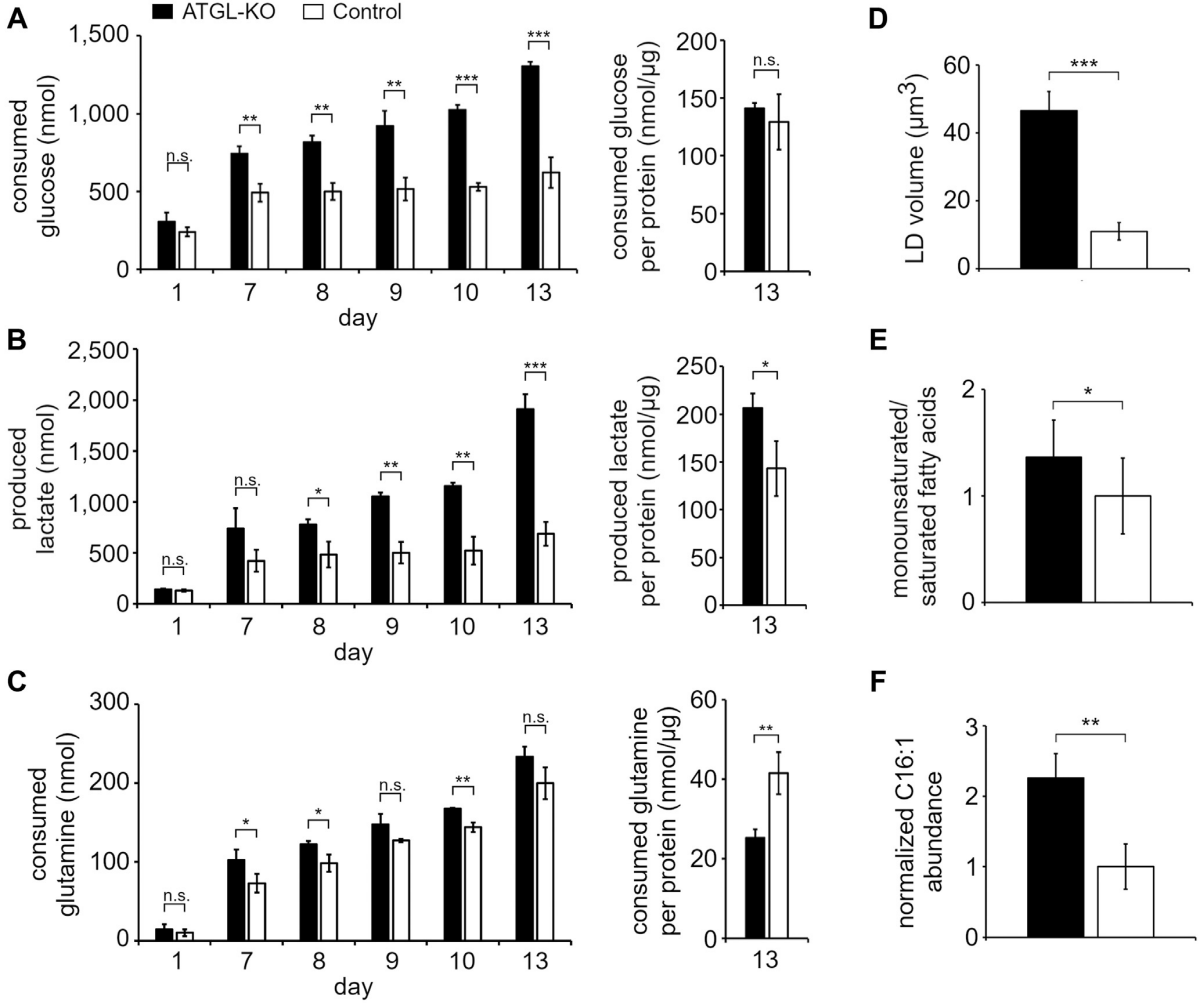
**ATGL-KO spheroids show higher glucose consumption/lactate excretion.***A*, glucose was assessed in culture supernatant by Glucose Glo Assay; glucose consumption was calculated from glucose concentration in original medium minus glucose concentrations of medium after indicated days of culture. *B*, lactate in the medium was assessed by Lactate Glo Assay. *C*, glutamine was assessed in the medium by Glutamine Glutamate Glo Assay; glutamine consumption was calculated from glutamine concentration in the original medium minus glutamine concentrations of medium after indicated days of culture. n = 3 biological replicates in each group; data from two independent experiments are shown. Spheroids in this representative experiment were cultured for 13 days, and protein quantitation used for normalization was performed on day 13. *D*, LD volume was assessed in cells after spheroid dissociation (n = 3 biological replicates in each group; n = 76 (ATGL-KO) or n = 80 (Control) cells in total). *E*, the ratio of MUFAs (C16:1 and C18:1) to SFAs (C16:0 and C18:0) was calculated based on FAME measurements and normalized to control (value of control group set to 1; n = 3 biological replicates in each group). *F*, the abundance of C16:1 FAME was normalized to an internal standard and protein abundance (value of control group set to 1; n = 3 biological replicates in each group); ns, not significant; ∗*p* < 0.05; ∗∗*p* < 0.01; ∗∗∗*p* < 0.001. ATGL, adipose triglyceride lipase; ATGL-KO, ATGL knockout; FAME, fatty acid methyl ester; LD, lipid droplet; MUFA, monounsaturated fatty acid.

To functionally validate higher influx of glucose and potential tendency toward a more pro-Warburg phenotype upon loss of ATGL, we carried out an *in vitro* glucose consumption/lactate production assay in spheroid (3D) and 2D culture. As expected, ATGL-KO spheroids were depleting the glucose from the media at a much faster rate, simultaneously producing higher quantities of lactate compared with control cells (Fig. 4, *A* and *B*). While lactate production was still significantly higher in ATGL-KO spheroids when normalized on protein amount per spheroid, glucose consumption was not significantly different between ATGL-KO and control spheroids. This may suggest that cells in the core of the spheroid, despite higher expression of GLUTs and related enzymes, were no longer able to take up extracellular glucose because of a lack of vascularization. It has been proposed that spheroids have a highly proliferating outer rim, which makes them grow at high rates, whereas the inner cells are quiescent or necrotic (26). Our histological analysis of the spheroids is in line with these findings because the spheroids clearly depict more proliferation marker in the outer rim (Fig. 2*B*). We observed that contrary to the compact and round controls, ATGL-KO spheroids grew more “loosely”, with less condensed core and higher spread of the outer, highly proliferating layer (Fig. 2*B*). This phenotype could resemble a coping mechanism to maximize nutrient access in the lack of vascularization. While ATGL-KO cells showed a higher consumption of glucose and a higher production of lactate (normalized to protein amount) as multicellular tumor spheroids, we found no significant difference in lactate production and glucose consumption when the same cells were cultured in 2D (supplemental Fig. S4, *D* and *E*), suggesting that the observed more glycolytic phenotype is really a characteristic of ATGL-KO spheroids. The possibility that the high lactate levels in ATGL-KO spheroids arise because of high numbers of dead cells in the core was excluded after dead-cell stain revealed very low numbers of dead cells in both ATGL-KO and control spheroids (supplemental Table S4; supplemental Fig. S3).

Because glucose consumption in control spheroids did not seem to increase over a period of days (Fig. 4*A*), we hypothesized that control cells might rely on a different nutrient source. Recent reports highlight that hypoxia can, in addition to glucose, also boost glutamine uptake of cancer cells (42). Indeed, when glutamine consumption was assessed, we could see that while the total consumption of glutamine was higher for ATGL-KO spheroids, the overall rate of consumption was not very different from the controls (Fig. 4*C*). When normalized on protein content, it became apparent that control cells took up proportionally more glutamine than ATGL-KO cells (Fig. 4*C*). These data suggest that while ATGL-KO cells primarily lived on glucose, the control cells were much more glutamine-dependent.

Besides the measurement of important metabolites of glucose metabolism, LD and FA analysis was performed to support the observed proteomic changes. Like previously reported for A549 ATGL-KO and control cells grown in 2D (19), the LD volume was significantly higher in spheroid-derived ATGL-KO cells compared with the spheroid-derived controls (Fig. 4*D*). Moreover, an increased ratio of MUFA to saturated fatty acids as well as higher levels of C16:1 fatty acid (Fig. 4, *E* and *F*) was detected in ATGL-KO spheroids. This is in accordance with the observed increased abundance of FADS2, which catalyzes the conversion of saturated fatty acids (preferentially C16:0 FA) into MUFAs (such as C16:1 FA).

### Increased Growth and Altered Proteome of ATGL-KO A549 Spheroids Grown on Chorioallantoic Membranes

**Fig. 5:**
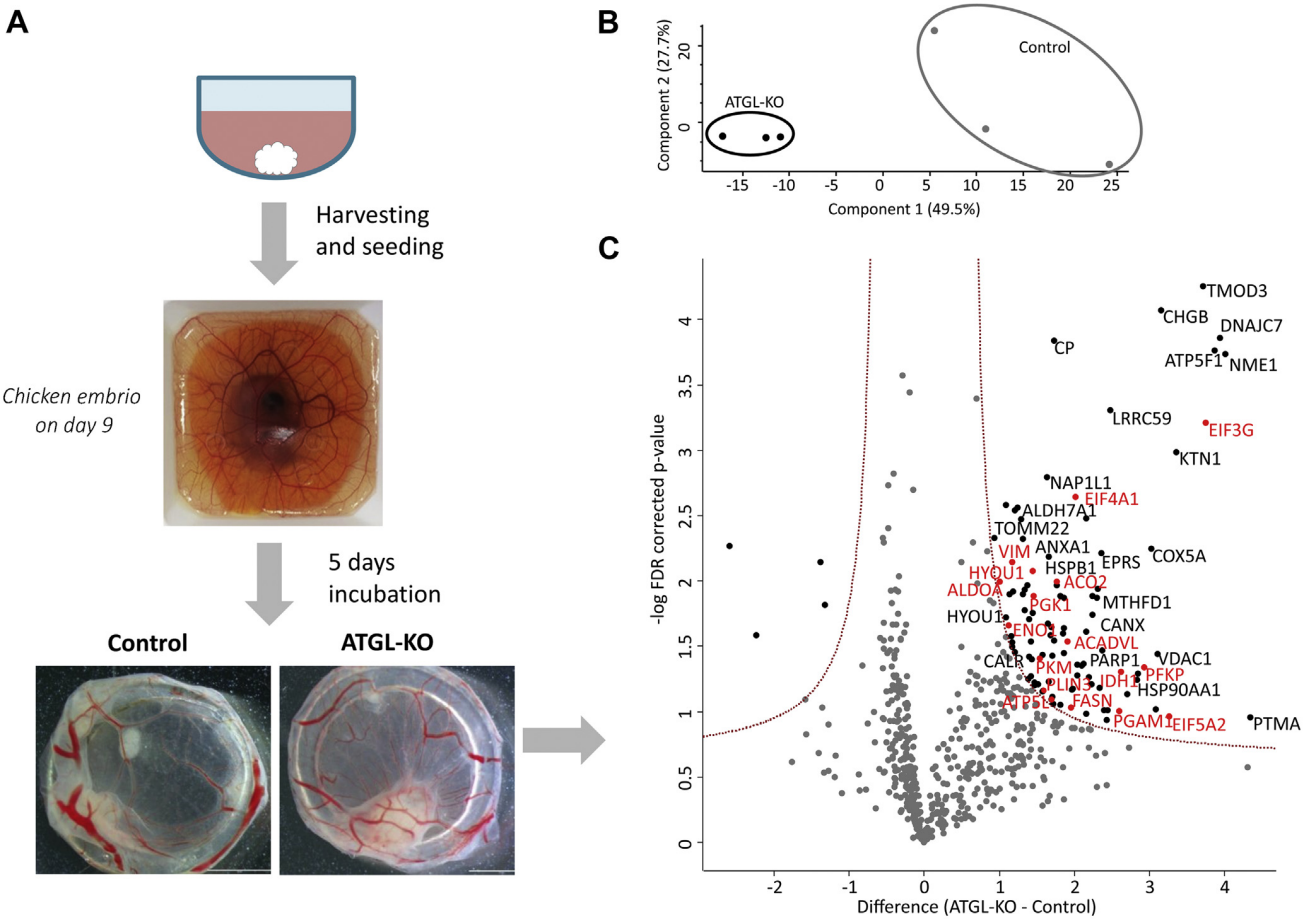
**3D cultured ATGL-KO A549 cells grown on CAM form bigger tumors and display proteomic changes**. *A*, CAM assay workflow. *B*, principal component analysis of ATGL-KO and control samples. *C*, volcano plot of the LFQ data after filtering for at least three valid values in at least one group; significance threshold Student *t* test *p*-value 0.05, FDR 5%, S0 0.5; proteins marked in *red* are addressed in more detail in the text. ATGL, adipose triglyceride lipase; ATGL-KO, ATGL knockout; CAM, chorioallantoic membrane; FDR, false discovery rate; LD, lipid droplet; LFQ, label-free quantitation.

To provide the spheroids with an even more *in vivo*-like environment and overcome potential pitfalls because of lack of tumor microenvironment, we carried out the chick CAM assay. The CAM assay is a robust technique used for a plethora of cancer studies, from angiogenesis to invasion and metastasis (22, 43). For this purpose, we again generated spheroids from ATGL-KO and control A549 cells over the period of 10 days, after which we transferred the spheroids onto the surface of CAM and allowed them to grow for another 5 days before harvesting (Fig. 5*A*).

**Fig. 6:**
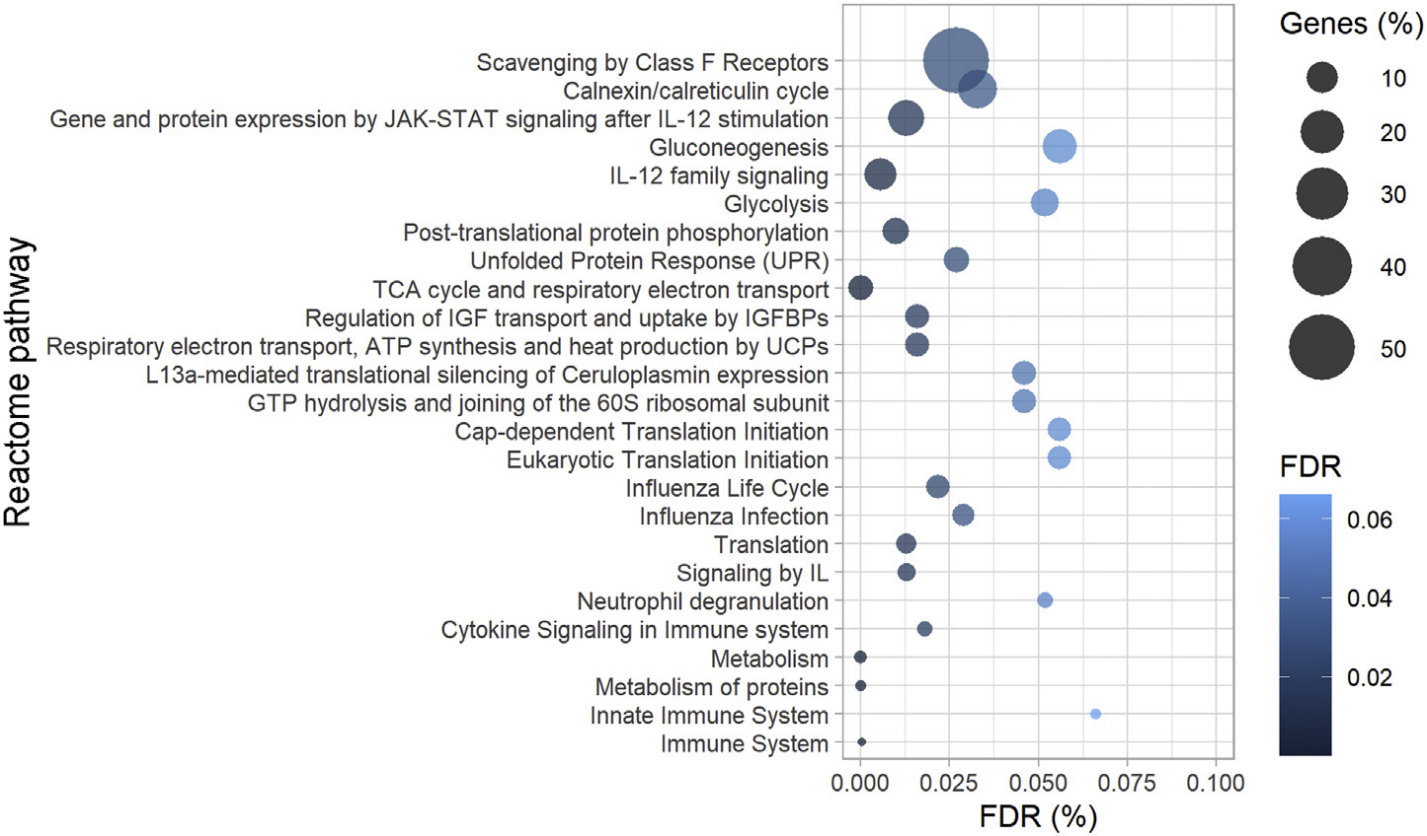
**Highly enriched pathways in ATGL-KO spheroids grown on CAM.** Proteins up-regulated at least 2-fold were included in the String analysis. The top 25 most enriched Reactome pathways are shown. Percentage of genes (Genes (%), illustrated by the size of the *circle*) represents the proportion of matching between the protein list used as input and the background (human proteome). Color of the circle illustrates the false discovery rate (FDR) corrected *p*-value (in percent; *darker color* means higher significance). BP, binding protein; CAM, chorioallantoic membrane; GTP, guanosine triphosphate; IGF, insulin-like growth factor; IL, interleukin; TCA, tricarboxylic acid; UCP, uncoupling proteins.

Spheroid derived tumors were then also subjected to LFQ protein analysis. As visible already by principle component analysis, clusters of ATGL-KO and control samples separated well (Fig. 5*B*), suggesting prominent differences in protein expression profiles between the two groups (Fig. 5*C*, supplemental Table S2). ATGL-KO spheroid-derived tumors showed many more upregulated proteins compared with control (Fig. 5*C*, supplemental Table S2). Significantly upregulated proteins in the ATGL-KO spheroid-derived tumors with a fold change larger than two were subjected to Reactome pathway analysis (Fig. 6; supplemental Table S3).

We again observed a prominent upregulation of proteins involved in glucose metabolism, glycolysis, and protein translation. Furthermore, we observed an upregulation of interleukin signaling pathways (such as interleukin 12). Interleukin-12 signaling is suggested as a promising candidate for tumor immunotherapy with potential antiangiogenic effects (44, 45).

Among the significant FDR-corrected proteins with higher abundance in ATGL-KO spheroids, we identified several glycolytic proteins (*PFKP*, Q01813; *ALDOA*, P04075; *PGK1,* P00558; *PGAM1*, P18669; *ENO1*, P06733; *PKM*, P14618) as well as tricarboxylic acid (TCA) cycle proteins (aconitate hydratase, *ACO2*, Q99798; isocitrate dehydrogenase 1, *IDH1,* O75874) and proteins of the electron transport chain (ATP synthase subunit g, *ATP5L*, O75964), all indicating higher glucose consumption. In addition, several enzymes involved in lipid metabolism were found upregulated in ATGL-KO spheroids (fatty acid synthase, *FASN,* P49327; very-long-chain specific acyl-CoA dehydrogenase, *ACADVL*, P49748; Perilipin-3, *PLIN3*, O60664) as well as proteins involved in protein synthesis (eukaryotic translation initiation factor 3 subunit G, *EIF3G*, O75821; eukaryotic translation initiation factor 5A-2, *EIF5A2*, Q9GZV4; eukaryotic translation initiation factor 4A-1, *EIF4A1*, P60842). These proteins point toward a higher production of biomass as well as increased *de novo* FA synthesis, all of which corroborates a more pronounced cancer phenotype.

In this study, we could identify significant metabolic changes in lung cancer cells upon knockout of the major triglyceride-cleaving lipase ATGL. Previously, we reported of changes in this cell model when cultured as a monolayer (19). Strikingly, more *in vivo*-like culturing methods (3D and CAM) revealed a more aggressive cancer metabolic signature in ATGL-KO cells, which was heretofore not recognized.

## Discussion

It is widely accepted that cancer cells undergo prominent metabolic changes to meet their needs and support their proliferative (and aggressive) phenotype. In addition to the well-known changes in glucose metabolism, reprogramming of FA metabolism in cancer is crucial for keeping up with the increased need of FAs for biomembrane synthesis (5). The role of lipolysis in cancer metabolic changes is inconclusive, especially concerning the first enzyme of the lipolytic cascade—ATGL (19, 20, 46, 47, 48, 49, 50, 51, 52). Nowadays, 3D cell culture is often applied as alternative *in vitro* model, as numerous studies have shown that 3D cell culture better mimics the physiological conditions of solid tumors (21, 26, 53). In this study, we therefore revisited the role of ATGL in lung cancer cells employing 3D cell culture and CAM assay, both models used to approach conditions more similar to solid tumors and the tumor microenvironment. The comparison of growth of A549 ATGL-KO cells and their corresponding control cells revealed a more pronounced difference when cells were cultured as 3D spheroids or 3D spheroid–derived tumors (CAM assay) compared with a 2D monolayer. More strikingly, we found a prominent switch toward a more glycolytic pro-Warburg phenotype under these conditions. Unlike to monolayer growth, where cells are uniformly exposed to nutrients and oxygen, in 3D culture, a gradient of oxygen and nutrient availability is formed, resembling the formation of a solid tumor *in vivo* more closely. ATGL in particular seems to facilitate the metabolic adaptation to such a condition. As a result, many of the glycolytic enzymes and proteins involved in FA metabolism were upregulated upon knockout of ATGL when cells were grown in a 3D manner and especially when cultured on an *in vivo* representative tumor model (CAM).

Proteomics of A549 spheroids revealed upregulated enzymes of the glycolysis (*ADPGK*, Q9BRR6 and *PFKP*, Q01813) and glucose uptake (*GLUT1*, P11166) pathways upon ATGL knockout. In tumors derived from the same spheroids on CAM assay, an even higher number of enzymes of the glycolytic pathway were found upregulated: ATP-dependent 6-phosphofructokinase (*PFKP*, Q01813), fructose-bisphosphate aldolase A (*ALDOA*, P04075), phosphoglycerate kinase 1 (*PGK1*, P00558), phosphoglycerate mutase 1 (*PGAM1*, P18669), alpha-enolase (*ENO1*, P06733), and pyruvate kinase (*PKM*; P14618). In addition to glycolytic enzymes, fatty acid synthase (*FASN,* P49327) was higher abundant in ATGL-KO spheroid-derived tumors. Both higher rates of glycolysis and *de novo* FA synthesis are typical cancer characteristics (2, 5).

Interestingly, we also identified two enzymes of the tricarboxylic acid cycle: aconitate hydratase (*ACO2*, Q99798) and isocitrate dehydrogenase (*IDH1,* O75874) as well as the ATP-synthase subunit g (*ATP5L*, O75964) to be upregulated in spheroid-derived tumors from ATGL-KO cells. Owing to the highly glycolytic metabolism in cancer cells, it was long believed that mitochondria and thus mitochondrial pathways such as TCA cycle are defective. Recent findings, however, have shown that TCA cycle and mitochondrial metabolic pathways are crucial for the production of biomolecules and precursors (54) and that proliferating cells are in need of reduced carbon and nitrogen as well as reductive equivalents like NADPH (1). Interestingly, the here identified enzyme IDH1 was reported to enable the reductive formation of citrate in the cytosol, and this reaction occurs upon detachment from monolayer culture into anchorage-independent growth (*e.g.*, spheroids) (55). Produced citrate is then transported into and utilized within mitochondria, where it undergoes oxidative decarboxylation, yielding both alpha-ketoglutarate and reductive equivalents (NADPH) that mitigate mitochondrial reactive oxygen species stress (55). Researchers found that spheroid growth but not monoculture growth was reduced upon suppression of IDH1, which highlights the importance of this enzyme that we found upregulated in ATGL-KO spheroid-derived tumors for anchorage-independent growth (55). ATGL-KO spheroid derived tumors also show increased levels of the epithelial-to-mesenchymal transition marker vimentin (*VIM*, P08670). Vimentin is not only correlated with but required for lung cancer metastasis (56), which suggests increased aggressiveness of ATGL-KO spheroid derived tumors.

CAM assay was used to provide an even more *in vivo*-like model to study ATGL-KO in lung cancer cells. The thereby used CAM on which the spheroids are mounted is highly vascularized, which allows angiogenesis (57), a characteristic that is not possible in spheroids alone. Even though CAM-assay facilitates some degree of vascularization, the spheroid-derived tumors may still bear a hypoxic core similar to those of solid tumors because of their large size, especially in ATGL-KO spheroid-derived tumors. In line with this notion, we identified several proteins in those tumors that are usually upregulated in hypoxic conditions. One example is the heat-shock protein hypoxia upregulated protein 1 (*HYOU1*, Q9Y4L1), which is associated with tumor prognosis (58, 59) and higher abundant in ATGL-KO spheroid-derived tumors. Hypoxia upregulated protein 1 is thought to play an important cytoprotective role in hypoxia by preventing apoptosis-induced cell death (59), which could contribute to the increased growth of these tumors. The partial or complete unavailability of oxygen (and nutrients) is one of the main features that differs between the cell culture models that were used in this study (2D *versus* 3D/CAM). Hypoxia is a major characteristic of solid tumors that induces several changes in cancer metabolism regulated through a family of transcription factors: HIFs (60). Some well-characterized HIF-induced changes in glucose metabolism include the upregulation of GLUTs and glycolytic enzymes (11), which we also observed in ATGL-KO cells in the at least partially hypoxic cell culture models, but not in 2D cell culture. In addition to changes in glucose metabolism, HIFs regulate lipid metabolism, through upregulation of enzymes like FASN and activation of peroxisome proliferating-activated receptor γ signaling, which in turn increases FA uptake (61, 62). We found FASN (*FASN*, P49327) upregulated in both ATGL-KO spheroids and spheroid-derived tumors, suggesting higher *de novo* fatty acid synthesis. In the ATGL-KO cells, this is further coupled with accumulation of LDs as a consequence of ATGL loss (19). Accumulation of LDs is associated with both cancer and hypoxia (6, 16) and is suggested to protect against reactive oxygen species stress (63). Another LD-associated protein we found upregulated in ATGL-KO spheroid-derived tumors was Perilipin-3 (*PLIN3*; O60664). Perilipin-3 has been described to play a role in LD stabilization, which is crucial for cells with a high accumulation of LDs (64). FA metabolism is also altered by hypoxia without involvement of HIFs. Enzymes like SCD1 require oxygen for their function (12). Interestingly, we observed an alternative fatty acid desaturase (*FADS2,* O95864) upregulated in ATGL-KO spheroids. Vriens *et al.* (33) described that this desaturase provides an alternative in the synthesis of MUFAs, which is an important class of FAs for proliferating cells when the main desaturase SCD1 is inhibited.

Despite the numerous metabolic changes, we observe in A549 spheroids and spheroid-derived tumors, it is not evident how ATGL loss can lead to such pronounced metabolic changes in this model system. The most distinct difference between 2D and 3D cell culture is the availability of oxygen and nutrients for individual cells (21). While cells grown as a monolayer have easy access to nutrients and oxygen and display a different morphology, cells grown in a more complex 3D structure behave differently (65). It has to be noted that ATGL is actively inhibited by HILPDA in hypoxia (16, 18). This suggests that cells under hypoxia benefit from ATGL repression and by depleting the cells from ATGL completely they may have a natural growth benefit under hypoxia. Still, the question remains how ATGL loss causes these pronounced glucose metabolic changes especially in (partly hypoxic) spheroid and spheroid-derived tumor conditions.

Taken together, our results suggest that loss of ATGL contributes to cancer aggressiveness through enabling a metabolic switch toward glycolytic metabolism under *in vivo*-like conditions. Our work additionally highlights the importance of choosing adequate cell model systems for particular research questions, such as cancer metabolism, as many of our phenotypic observations were made in 3D and CAM but not 2D culture experiments.

## Data Availability

The mass spectrometry proteomics data have been deposited to the ProteomeXchange Consortium *via* the PRIDE (23) partner repository with the dataset identifier PXD021105 and https://doi.org/10.6019/PXD021105.

In addition, all spectral files are available for preview using MS Viewer (https://msviewer.ucsf.edu/) under following search keys: **octvhdisxq** for 3D spheroids dataset and **vaqbljjeke** for 3D spheroids on CAM dataset.

## Supplemental data

This article contains supplemental data.

## Conflict of interest

The authors declare that they have no known competing financial interests or personal relationships that could have appeared to influence the work reported in this paper.
