# Supplementary material for: Adipose Triglyceride Lipase Loss Promotes a Metabolic Switch in A549 Non–Small Cell Lung Cancer Cell Spheroids
Source: Mol Cell Proteomics. 2021 May 14;20:100095. doi: 10.1016/j.mcpro.2021.100095 (PMC8214150; doi:10.1016/j.mcpro.2021.100095)
Supplement: Supplemental Figures S1–S5 [file mmc5.pdf]

Adipose Triglyceride Lipase Loss Promotes a Metabolic Switch in A549 Non-Small Cell Lung Cancer Cell Spheroids

Sophie Honeder, Tamara Tomin, Laura Nebel, Juergen Gindlhuber, Katarina Fritz-Wallace, Maximilian Schinagl, Christoph Heininger, Matthias Schittmayer, Nassim Ghaffari-Tabrizi-Wizsy, Ruth Birner-Gruenberger

Material included

Supplemental Material and Methods

Supplemental Figures

Supplemental Tables (added as .xlsx files)

Supplemental Material and Methods

Growth curve 2D (supplement)

For assessment of 2D growth, 80,000 cells were seeded into the wells of a 12-well plate (VWR) in duplicates. Growth was analyzed through observation of the cells for up to 96 hours under a Zeiss Cell Observer microscope. Seven positions in each well were chosen and images were acquired hourly. Collected images were subjected to analysis by an analysis tool which counts the numbers of pixels that are occupied by the cells in each image. From the output of all the positions in one well a mean was calculated that was plotted for each timepoint. From the plot, linear slopes of the exponential growth phase were determined, which represent the different growth behavior.

Live and Dead Cell Estimation

Dissociated cells were stained with 1:1000 SYTOX™ Red dead-cell stain (Invitrogen, #S34859) for 10 min at 37°C. Subsequent flow cytometry was performed on a cytoFLEX LX flow cytometer and single cell count as well as the percentage of dead cells was assessed with the CytExpert software (version 2.3.1.22).

Supplemental Figures

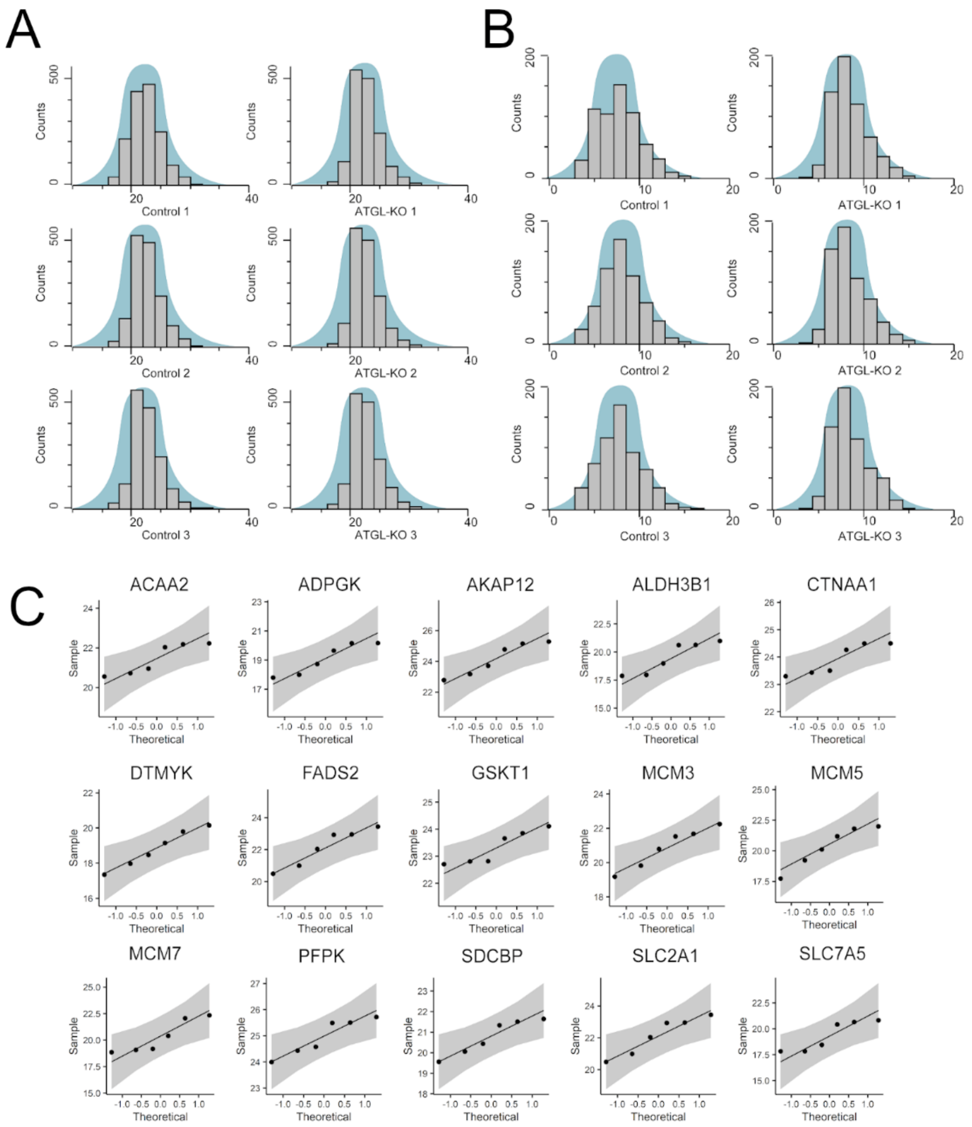

Figure S1. Proteomics data is normally distributed. A: Histograms of individual clones of 3D spheroids; B: Histograms of individual clones of 3D spheroids on CAM; C: quantile-quantile (qq) plots of selected proteins differentially regulated from 3D spheroid and 3D spheroid on CAM datasets.

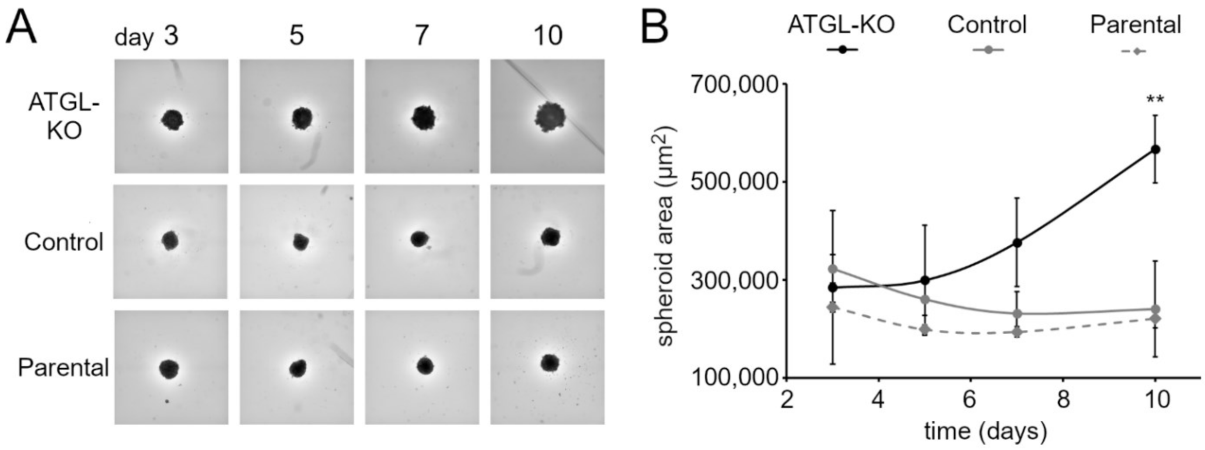

Figure S2. Spheroid growth curve. A: representative images of A549 ATGL-KO, control or parental spheroids on days 3, 5, 7 and 10 after seeding. B: growth curve of spheroids – mean spheroid area over time (n=3 biological replicates in ATGL-KO and control group; n=37-38 spheroids in ATGL-KO and control clones and n=32 A549 parental spheroids); ** $p < 0.01$

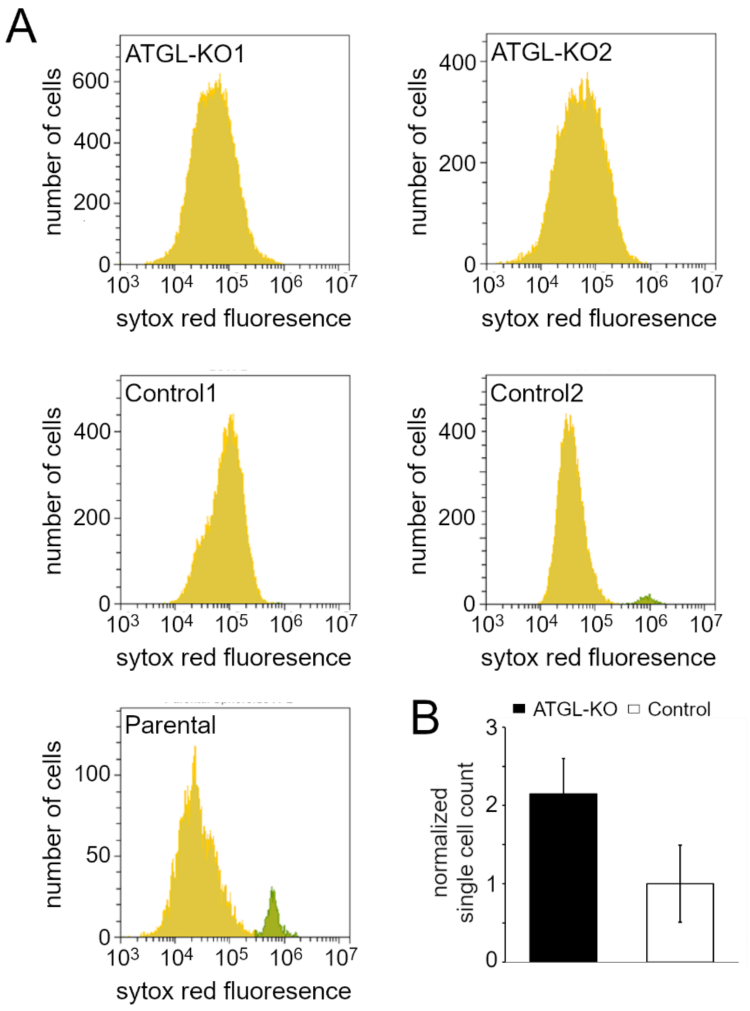

Figure S3. Dead cell stain of dissociated spheroids shows that spheroids contain mostly viable cells. A: A shift in sytox red fluorescence indicates the presence of dead cells in the single cell population of cells dissociated from spheroids. ATGL-KO as well as Control spheroids show a max. of 3.5% dead cells in the single cell population, while in parental spheroids this number is as high as 8.9%. B: number of single cells counted by flow cytometry, represented as single cell count and normalized to the mean of the control group (control is set to 1; n=3 biological replicates)

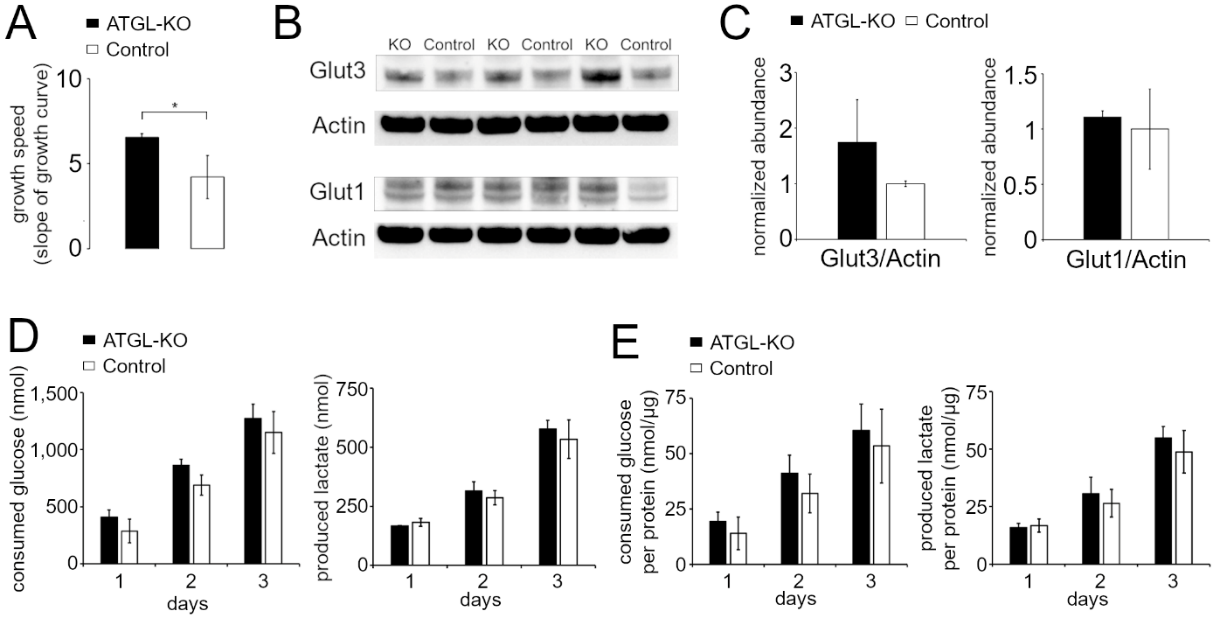

Figure S4. A549 ATGL-KO cells grown in 2D show less pronounced phenotype than when grown in 3D. A: Growth difference in A549 ATGL-KO and Control cells cultured as a monolayer (2D) in RPMI-1640 medium (2 g/L glucose), represented as slope of growth curve (by microscopy): area occupied by cells normalized to time 0 [%] over hours of growth analysis [h] (n=3 biological replicates). B: Western Blot of A549 ATGL-KO and control cells grown as a monolayer (2D). C: Quantification of bands from western blot (2D cells): represented as abundance normalized to the mean of the control group (control is set to 1; n=3 biological replicates). D: Glucose consumption/lactate excretion of A549 ATGL-KO and control cells cultured as a monolayer (2D) (n=3 biological replicates) E: Glucose consumption/lactate excretion normalized on protein content of cells harvested on day 3; * $p < 0.05$; ** $p < 0.01$; *** $p < 0.001$.

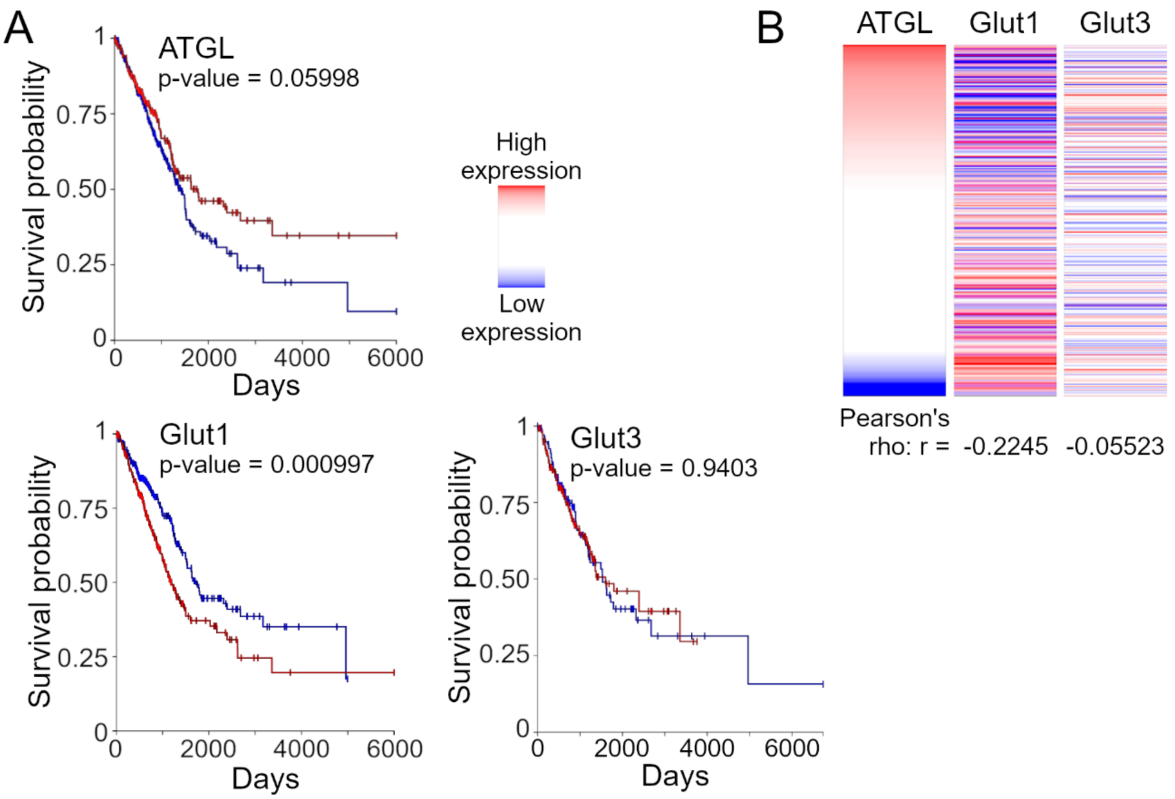

Figure S5. Higher ATGL correlates with lower Glut1 expression and better survival in a lung adenocarcinoma dataset (n=706). A: Kaplan-Meier plot of ATGL, Glut1 and Glut3 using a TCGA LUAD dataset. B: Gene expression correlation of ATGL with Glut1 and Glut3 on a lung adenocarcinoma dataset from TCGA (LUAD) – plots were generated using the Xena Functional Genomics Explorer.

Supplemental Tables

Added externally to this PDF as .xlsx files:

Suppl. Table S1. 3D proteomics – list of proteins for statistical analysis

Suppl. Table S2. CAM proteomics – list of proteins for statistical analysis

Suppl. Table S3. Reactome Pathway enrichment_3DonCAM_FC_KO to WT more than2

Suppl. Table S4. Flow Cytometry data_LiveDead_CellCount
